# Autonomic imbalance: prophet of doom or scope for hope?

**DOI:** 10.1111/j.1464-5491.2010.03184.x

**Published:** 2011-06

**Authors:** A I Vinik, R E Maser, D Ziegler

**Affiliations:** Eastern Virginia Medical School, Strelitz Diabetes Research Center and Neuroendocrine UnitNorfolk, VA; * Department of Medical Technology, University of DelawareNewark, DE, USA; † Institute for Clinical Diabetology, German Diabetes Center at the Heinrich Heine University, Department of Metabolic Diseases, University HospitalDüsseldorf, Germany

## Abstract

It has long been recognized that cardiac autonomic neuropathy increases morbidity and mortality in diabetes and may have greater predictive power than traditional risk factors for cardiovascular events. Significant morbidity and mortality can now be attributable to autonomic imbalance between the sympathetic and parasympathetic nervous system regulation of cardiovascular function. New and emerging syndromes include orthostatic tachycardia, orthostatic bradycardia and an inability to use heart rate as a guide to exercise intensity because of the resting tachycardia. Recent studies have shown that autonomic imbalance may be a predictor of risk of sudden death with intensification of glycaemic control. This review examines an association of autonomic dysregulation and the role of inflammatory cytokines and adipocytokines that promote cardiovascular risk. In addition, conditions of autonomic imbalance associated with cardiovascular risk are discussed. Potential treatment for restoration of autonomic balance is outlined.

## Introduction

Physiological activities of the cardiovascular system are under the control of the autonomic nervous system. Analysis of heart rate variability is a non-invasive electrocardiographic method for assessing overall autonomic activity. Analysis of heart rate variability, with analysis of respiratory activity, independently and simultaneously measures parasympathetic and sympathetic activity [1] and thereby provides information with regard to autonomic balance of the cardiovascular system. Autonomic balance involves complex interactions with several physiologic mechanisms that act to maintain heart rate and blood pressure within normal limits. Recent investigations have suggested that autonomic dysfunction (e.g. heightened activity of the sympathetic nervous system and suppressed activity of the parasympathetic nervous system) impairs the ability of the autonomic nervous system to regulate the cardiovascular system. New and emerging syndromes include orthostatic tachycardia, orthostatic bradycardia and an inability to use heart rate as a guide to exercise intensity because of the resting tachycardia. With a growing understanding of bi-directional interactions between the sympathetic and parasympathetic efferent pathways at different levels of the neuraxis and at target organs [2], it is possible that autonomic imbalance may be shown to be a key component involved in both the aetiology and the clinical course of cardiovascular disease.

## Morbidity and mortality

Cardiac autonomic neuropathy defined by measures of abnormalities of the parasympathetic and sympathetic nervous systems is a significant cause of morbidity and mortality associated with a high risk of cardiac arrhythmias and sudden death, possibly related to silent myocardial ischaemia. Cardiovascular events are the main cause of excess mortality among patients with both Type 1 and Type 2 diabetes [3]. The prevalence of cardiac autonomic neuropathy varies greatly depending on the criteria used to identify cardiac autonomic neuropathy, the assessment modalities and the patient cohort studied. These range from as low as 1.6 to 2.6% of the primary prevention cohort in the Diabetes Control and Complications Trial (DCCT) [4] to as high as 90% of patients with long-standing Type 1 diabetes who were potential candidates for a pancreas transplant [5]. In a large cohort of patients with Type 1 and Type 2 diabetes, Ziegler and colleagues, using predefined heart rate variability and spectral analysis of the R-R intervals, found that 25.3% of patients with Type 1 diabetes and 34.3% of those with Type 2 diabetes had abnormal findings [6]. Even the pre-diabetic stage (i.e. impaired glucose tolerance) is associated with a decreased parasympathetic modulation of the heart and a shift toward augmented sympathetic tone. Thus, parasympathetic tone may decline with an autonomic imbalance shifting toward augmented sympathetic tone during the development from normal glucose tolerance to impaired glucose tolerance and finally diabetes [7].

Reduced heart rate variability as a marker of autonomic dysfunction has been shown to have dire consequences in terms of morbidity (e.g. progression of coronary atherosclerosis) and mortality [8], independent of cardiovascular risk factors in various populations, including those with pre-diabetes and diabetes [9] [10]. In Type 1 diabetes, there is a fourfold increased risk of death [11]. Clearly the role of autonomic imbalance and the mechanisms underlying the risk require further exploration if we are to achieve benefit for all.

### Is hyperglycaemia and its control the solution to the impact of autonomic imbalance on morbidity and mortality?

The beneficial effect of glycaemic control on heart rate variability shown in the Epidemiology of Diabetes Interventions and Complications (EDIC) study [12] waned after several years [13]. In Type 2 diabetes, the benefit of multifactorial risk intervention after 7.8 years could be retained after a further observation period of 5.5 years, as shown in the Steno Trial [14] [15]. In contrast, in the Veterans Affairs Diabetes Trial (VADT), a trend toward an increase in incidence of autonomic neuropathy was noted in patients on intensive diabetes therapy as compared with those on standard therapy [16]. The concept that historical glycaemic control is a major determinant of diabetes control, known as metabolic memory or the ‘legacy effect’, has recently been reported for autonomic neuropathy in the Epidemiology of Diabetes Interventions and Complications study [12]. In the Diabetes Control and Complications Trial, intensive glycaemic control achieved a reduction of HbA_1c_ of 1.7%, (5 mmol/mol), that over 6.5 years was associated with a 54% reduction in the incidence of cardiac autonomic neuropathy when compared with conventional treatment. At 13 years’ follow-up, HbA_1c_ was similar in the originally randomized groups, yet the intensively treated group still enjoyed a 30% lower rate of cardiac autonomic neuropathy. This suggests a metabolic memory for the impact of hyperglycaemia [17]. It is thought that metabolic memory is the result of long-term molecular changes, such as flux through the polyol and hexosamine pathways and activation of protein kinase C [18], as well as oxidative and nitrosative stress [19]. This suggests that therapies might be directed at some of these downstream events [20]. Epidemiologic data strongly support the role of cardiovascular risk factors such as smoking, blood pressure and hyperlipidaemia in the development of cardiac autonomic neuropathy [21] [22], and clinical and basic research emphasize the role of these disturbances in function [23]. The final common pathway may include endothelial dysfunction, inflammation, cytokine overproduction and growth factor deficiency [1].

### Are inflammatory cytokines and adipocytokines associated with autonomic imbalance?

Interleukin 6 is a multifunctional cytokine that plays a central role in inflammatory responses. There is also evidence for the expression and activity of interleukin 6 in the nervous system. C-reactive protein is an acute-phase reactant produced in the liver in response to interleukin 6. These inflammatory markers, C-reactive protein and interleukin 6, were found to be associated with reduced heart rate variability in a study of 264 middle-age male twins free of symptomatic coronary artery disease [24]. The results of this cross-sectional study suggest that autonomic dysregulation may lead to inflammation providing a pathway through which traditional risk factors promote the development of cardiovascular disease. It has been hypothesized that inflammation alters heart rate variability, but the opposite directional relationship suggesting that autonomic changes would be pro-inflammatory is also conceivable [24]. Experimental data support both possibilities. Both exposure to acetylcholine and direct vagal stimulation inhibits release of cytokines by macrophages [25]. Conversely, sympathetic activation is pro-inflammatory. In isolated adipocytes, β-stimulation increases interleukin 6 [26], whereas β-blockers dampen the interleukin 6 increase normally seen in response to stress in rats [27]. Given that cause–effect relationships cannot be determined from cross-sectional studies, prospective studies will be needed to determine whether autonomic dysfunction mediates the inflammatory process or if autonomic imbalance is a marker of inflammation. An association between both C-reactive protein and interleukin 6 and diabetic polyneuropathy has also recently been demonstrated [28].

Hyperleptinaemia may be an important player in the activation of the sympathetic nervous system in human obesity. Leptin, a product of the obesity gene, is secreted from adipocytes and acts on the hypothalamus in the regulation of feeding and energy balance. Intravenous infusion of leptin increases arterial blood pressure and heart rate [29]. Adrenergic blockade abolishes the effects of leptin, suggesting that the effects of leptin were mediated by the sympathetic nervous system. In humans, leptin levels were associated with an increase in the low-frequency/high frequency-ratio (i.e. a shift of sympathovagal balance toward increased sympathetic activation) independent of anthropomorphic measures as shown in 120 non-obese adults [30].

Further support for a role of leptin in autonomic dysfunction derives from the report by Murialdo *et al*. that bulimia nervosa patients have altered heart rate variability associated with low leptin levels and apparent sympathetic insufficiency [31]. The weight-loss state is associated with profound effects on leptin levels, as well as autonomic function which strive to alter metabolic balance in favour of weight regain. With the administration of leptin, energy expenditure, skeletal muscle work efficiency and sympathetic tone, as well as levels of thyroxine and triiodothyronine, are restored toward levels prior to weight loss. Thus, the weight-reduced state may be regarded as leptin insufficiency, potentially correctible with leptin administration [32].

Leptin receptor-deficient *db/db* mice develop Type 2 diabetes, hypertension and obesity, with a disrupted circadian blood pressure. They also have higher resting heart rates and greater heart rate responses to α-adrenergic agonists and β-adrenergic blockers. However, blunted responses to atropine and decreased baroreceptor responses, as well as loss of heart rate variability, were also shown. Stimulation of central α-2 adrenoreceptors changed parasympathetic heart rate control and baroreceptor sensitivity. Thus, the authors suggest that *db/db* mice exhibit features found in humans with Type 2 diabetes and autonomic imbalance and could serve as a model for further study. Whatever transpires, it cannot be denied that there are important consequences of alterations in leptin homeostasis with those of balance of the autonomic nervous system [33].

Another adipocyte-derived protein that may be regulated by the sympathetic nervous system is adiponectin. In a mouse model, the sympathetic nervous system has been shown to regulate adiponectin levels and its synthesis in white adipose tissue *in vivo* [34]. In humans with autonomic imbalance (i.e. predominant sympathetic activation), there were low levels of circulating adiponectin [35] (i.e. a negative correlation between adiponectin levels and the low-frequency/high-frequency ratio). The low-frequency/high-frequency ratio was an independent determinant of adiponectin levels (*R*^2^ = 0.617). Adiponectin depolarizes parvocellular paraventricular nucleus neurons controlling neuroendocrine (ACTH not thyroid-stimulating hormone) levels and acts in the hypothalamus paraventricular nucleus to coordinate neuroendocrine and autonomic function through its actions on specific functional groups of paraventricular nucleus neurons [36]. In addition, adiponectin acts in the nucleus of the solitary tract to reduce blood pressure by modulating the excitability of neuropeptide Y neurons [37], but may also lower blood pressure by direct inhibition of the renal sympathetic nervous system [38]. Parasympathetic input to adipose tissue has been demonstrated by Kreier *et al*. [39], illustrating that white adipose tissue does receive dual autonomic control. This is important in the regulation of cytokine release, as well as the control of the release of free fatty acids and the development of oxidative/nitrosative stress. Thus, it is possible, as suggested by Kreier *et al*. [39], that an unbalanced autonomic nervous system outflow to organs within the intra-abdominal compartment (e.g. pancreas, liver, adipose tissue) may be a factor in the development of diseases associated with intra-abdominal obesity. There appears to be a closed-loop system, wherein the adipose tissue mass regulates the hypothalamic autonomic system, with both the sympathetic nervous system and parasympathetic nervous system impacting the metabolic and inflammatory potential of adipose tissue. Although the effects of adiponectin on sympathetic nervous system control have not been studied, clearly the reduction of inflammation, the inhibition of free fatty acid release and the attendant oxidative/nitrosative stress may be potential targets for future therapeutic endeavours.

Growing evidence suggests that enhanced oxidative/nitrosative stress and, in particular, increased production of the potent oxidant peroxynitrite (a product of superoxide anion radicals with nitric oxide) is a characteristic feature of both experimental and clinical diabetes mellitus [40]. Peroxynitrite causes damage to a variety of tissues by diverse effects, which include: nitration and nitrosylation of protein; damage to DNA; altered gene expression and changes in transcriptional regulation and signal transduction; altered mitochondrial function; and induction of microvascular endothelium necrosis in a variety of tissues [40] [41] [42], including peripheral nerve, spinal cord, dorsal root ganglion neurons and vasa nervorum of several different models of both Type 1 and Type 2 diabetes [43]. Several markers of oxidative stress in plasma, including superoxide and peroxynitrite, are elevated in patients with diabetes with cardiac autonomic neuropathy [44].

These findings suggest the presence of peroxynitrite cytotoxicity at both early and advanced stages of Type 1 and Type 2 diabetes and, furthermore, at the pre-diabetic stage. Enhanced nitrosative stress has also been documented in the circulation [45] and cutaneous microvasculature of human subjects with diabetes mellitus [46]. Monocyte nitrosylated protein expression is a new biomarker of metabolic control and inflammation in individuals with diabetes with macroangiopathy and correlates with measures of inflammation such as C-reactive protein [47]. Moreover, cyclo-oxygenase-2 activation appears to play an important role in the development of cardiac autonomic neuropathy, as cyclooxygenase-2 gene inactivation is protective against indexes of cardiac autonomic neuropathy, oxidative stress and inflammation and prevents left ventricular dysfunction and myocardial fibrosis in experimental diabetes [48].

A more detailed assessment of diabetic microvascular complications and autonomic function is needed to determine if this variable can be employed as a biomarker of the presence, severity and progression of diabetic autonomic neuropathy and accounts in part for the relatively low risk conferred by hyperglycaemia alone (Diabetes Control and Complications Trial and Epidemiology of Diabetes Interventions and Complications study) and the greater risk reduction for autonomic neuropathy with multiple risk factor reduction [14].

### Conditions of autonomic imbalance associated with cardiovascular risk

#### Metabolic syndrome

In a study of healthy individuals, prolonged mild hyperinsulinaemia was shown to disrupt the circadian rhythm of cardiac autonomic activity. Thus, the authors suggested that early changes in the neural control of cardiac activity may provide a potential mechanism mediating a pathophysiological link between impaired glucose tolerance and cardiovascular disease [49].

Individuals with the metabolic syndrome have alterations in the function of the autonomic nervous system as increased activity of the sympathetic nervous system is associated with several of the specific metabolic syndrome components (e.g. obesity, hypertension, insulin resistance) [50] [51]. Unresolved, however, is whether the aberrations of the autonomic nervous system contribute to the development of the metabolic syndrome or are a consequence of the metabolic syndrome. A recent study of 1298 individuals with different numbers of metabolic abnormalities showed that altered cardiac autonomic function existed in individuals with one or two metabolic abnormalities. Furthermore, as a result of cardiac autonomic dysfunction, but not insulin resistance in persons with one metabolic abnormality, the authors suggested that altered cardiac autonomic function precedes the presence of insulin resistance in the metabolic syndrome [52]. Prospective studies are, however, needed to answer this question with regard to the natural history. One prospective study of 433 non-obese, normotensive men followed for 5 years showed that autonomic dysfunction contributed to the development of obesity as sympathetic overactivity (i.e. plasma norepinephrine concentrations) and serum uric acid levels predicted future weight gain and elevation of blood pressure [53].

In individuals with a history of diabetes, it is well known that dysfunction of the autonomic nervous system is a potential complication. Impaired autonomic function may, however, be a mechanism associated with early glucose dysmetabolism and thus autonomic dysfunction may be involved in the pathogenic pathway leading to the development of diabetes [54].

#### Sleep apnoea

In obstructive sleep apnoea there is repetitive collapse of the upper airway during sleep. This leads to hypopnoea or apnoea with corresponding oxygen desaturation. The pathophysiology of obstructive sleep apnoea is multifactorial. Anatomical contributions of the upper airway (e.g. fat deposits altering pharyngeal size), as well as impaired peripheral/central control of muscles via alterations to the autonomic nerve fibres involved in the upper airway reflexes, may contribute to the pathogenesis [55]. There are significant changes in sympathetic/parasympathetic balance during the different stages of sleep (Table 1) [56]. Note the increase in low frequency and low-frequency/high-frequency ratio in rapid eye movement sleep and the decrease in high frequency (Table 1). Clearly, disturbances in sleep architecture, such as obstructive sleep apnoea or even changing duration of sleep can contribute significantly to a greater derangement in this balance, with large consequences on metabolic function.

**Table 1:** Autonomic functions in different sleep stages

HRV	Wakefulness	Stage 2	REM	Significance
TF	6.5 ± 6.2	6.3 ± 5.8	11.2 ± 10.2	0.001
LF	2.0 **±** 2.8	2.1 **±** 2.6	2.7 **±** 3.0	0.013
HF	1.2 **±** 1.5	1.7 **±** 2.4	1.3 **±** 2.7	0.012
LF/HF	2.7 **±** 2.6	2.0 **±** 2.2	3.9 **±** 3.6	0.001

HF, high frequency; HRV, heart rate variable; LF, low frequency; REM, rapid eye movement; TF, total frequency.

The prevalence of obstructive sleep apnoea in the diabetic population varies from 17% in one study of men with Type 2 diabetes [57] to 30% in non-obese subjects with diabetes who had diabetic autonomic neuropathy [58]. Differences in prevalence rates vary as a result of methodology issues, including different patient cohorts and different criteria used to define the severity of the hypopnoea or apnoea events.

Individuals with obstructive sleep apnoea have increased levels of sympathetic activity [59] [60], resulting in autonomic nervous system imbalance, potentially predisposing them to the development of cardiac arrhythmias [61]. Thus, obstructive sleep apnoea appears to be associated with adverse cardiovascular outcomes [62]. In one study, the risk of sudden death from cardiac causes for patients with obstructive sleep apnoea during the sleep hours of 00.00 to 06.00 h was shown to be 2.57 [63]. Obstructive sleep apnoea is associated with altered glucose metabolism and inflammation [64]. Abnormal sleep times [short (< 6 h) or long (> 8 h)] are related to increased risk of Type 2 diabetes [65]. Although the potential mechanisms for the increased risk of sudden death are many, obstructive sleep apnoea in patients with diabetes complicated by autonomic imbalance represents increased risk for cardiovascular morbidity and mortality [55]. Treatment of obstructive sleep apnoea with continuous positive airway pressure therapy has been shown to improve vagal tone [66]. Four weeks of continuous positive airway pressure reduced mean 24-h heart rate in men with moderate-to-severe obstructive sleep apnoea, possibly because of reduced sympathetic activation [67]. Whether continuous positive airway pressure may improve cardiac autonomic neuropathy or reduce cardiac arrhythmias in association with obstructive sleep apnoea in patients with diabetes requires further study.

### Impact of hypoglycaemia on autonomic function

Prior hypoglycaemic episodes attenuate the response of the autonomic nervous system to subsequent hypoglycaemia [68] and to specific cardiovascular stresses [69]. Given that antecedent hypoglycaemia also attenuates cardiovascular autonomic control, it could have implications with regard to the use of rigorous glycaemic control in individuals with diabetes. Adler *et al*. [69] suggested that the impact of antecedent hypoglycaemia on cardiovascular autonomic function could be a potential explanation for the increase in cardiovascular mortality reported in the Action to Control Cardiovascular Risk in Diabetes (ACCORD) study [70]. However, no association between antecedent hypoglycaemia and increased mortality in the ACCORD trial was shown, although cardiac autonomic neuropathy clearly predicted events [71].

### Distinguishing cardiac autonomic neuropathy from autonomic imbalance

The traditional view of cardiac autonomic neuropathy is that there is an early phase of loss of parasympathetic function with increased resting heart rate and abnormalities in the expiration:inspiration ratio of heart rate variability. There may, however, be no parasympathetic denervation as such, but simply early augmentation of sympathetic tone. Early in the natural history of diabetes there is impairment of parasympathetic function, with a relative increase of sympathetic function causing an imbalance of the sympathetic/parasympathetic tone. Later, sympathetic denervation follows, beginning at the apex of the ventricles and progressing towards the base of the heart [1], leading to yet another imbalance, with an increase in propensity to dysrhythmias.

**FIGURE 1:**
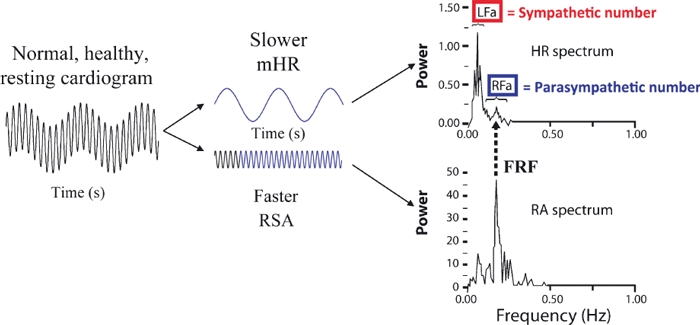
Parasympathetic and sympathetic measurement model. FRF, fundamental respiratory frequency; HR, heart rate; Lfa, low frequency area; Lfa/Rfa, sympathovagal balance; mHR, mean heart rate; RA, respiratory activity; Rfa, respiration frequency area; RSA, respiratory sinus arrhythmia.

Analysis of heart rate variability coupled with analysis of respiratory activity provides a non-invasive and objective method for assessing cardiac autonomic neuropathy and may be derived from electrocardiograph recordings [1]. Incorporating respiratory signal analysis enables one to independently measure each branch of the autonomic nervous system. Spectral analysis of heart rate variability is an important tool to evaluate cardiac autonomic neuropathy [1]. It decomposes a series of sequential R-R intervals into a set of sinusoidal waves. The power spectrum is displayed with the magnitude of variability as a function of frequency. The main frequency components are: very-low-frequency components (< 0.04 Hz) related to fluctuations in vasomotor tone associated with thermoregulation, the low-frequency component (0.04–0.15 Hz) associated with the baroreceptor reflex and the high-frequency components (0.15–0.4 Hz) related to respiratory activity [1]. The cardiogram (from the electrocardiograph) only provides one number (heart rate variability) for a two-part system (i.e. parasympathetic and sympathetic). If the one number (heart rate variability) changes, one cannot tell which part (parasympathetic or sympathetic) changed. Respiratory analysis adds the second number, identifying the parasympathetic activity that generates respiratory sinus arrhythmia, thereby enabling respiratory sinus arrhythmia to be analysed separately to identify parasympathetic activity (i.e. respiration frequency area) (Fig. 1). Heart rate variability which is mixed (parasympathetic and sympathetic) can now be separated (i.e. respiration frequency area and low frequency area) (Fig. 1).

## Future directions in restoration of autonomic balance and the impact on morbidity and mortality

### Lifestyle changes to restore balance

In the Diabetes Prevention Program, therapeutic lifestyle changes which included appropriate diet and exercise induced a 25% reduction in risk of autonomic dysfunction [72].

Endurance training improves heart rate variability in patients with minimal abnormalities. However, one cannot use the normal calculations for heart rate of 220 minus age to calculate the maximum intensity and to derive a target for intensity of exercise, because of the resting tachycardia in patients with autonomic dysfunction. Therefore, individuals must rely on use of perceived exertion to prescribe exercise intensity in diabetic autonomic neuropathy [73]. Chronic exercise is associated with enhanced cutaneous blood flow in Type 2 diabetes [74], restoration of baroreceptor sensitivity [75], as well as improved vagal activity and exercise capacity after 12 weeks of endurance training in early cardiac autonomic neuropathy, but not severe cardiac autonomic neuropathy [76]. These results emphasize the need for early aggressive intervention at the stage of physiological deficits in nerve function. Perhaps the most enlightening study was that of Motooka *et al*. [77], who showed that older people walking their pet animal improved sympathetic/parasympathetic balance, whereas the same exercise without the animal was quite stressful, increasing the prevalence of low frequency amplitudes, indicating loss of balance and sympathetic activation.

### Medications to restore sympathovagal balance

**FIGURE 2:**
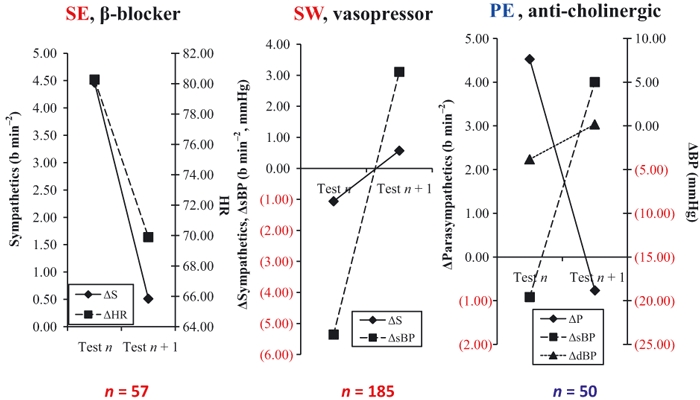
The ability to correct sympathetic excess with a β-adrenergic blocking agent. Δ, sympathetic withdrawal with an adrenergic agonist and parasympathetic excess with an anti-cholinergic agent [80]. BP, blood pressure; HR, heart rate; PE, parasympathetic excess; SE, sympathetic excess; SW, sympathetic withdrawal.

Disturbances in autonomic balance can arise from an increase or decrease in either sympathetic or parasympathetic tone. Each can be restored to normal using appropriate medications (Fig. 2).

The use of α-lipoic acid [78], a powerful antioxidant, has been shown to improve heart rate variability. The role for reactive oxygen species in diabetes-associated nerve blood flow and conduction deficits was demonstrated in studies with the ‘universal’ antioxidant DL-α-lipoic acid, which combines free radical and metal chelating properties with an ability (after conversion to dehydrolipoic acid) to regenerate levels of other antioxidants. Pharmacological tools targeting peroxynitrite formation or promoting its decomposition have recently been examined for their effects on autonomic neuropathy. Several peroxynitrite decomposition catalysts have been employed for preclinical studies of early peripheral diabetic neuropathy and autonomic neuropathy. The peroxynitrite decomposition catalysts Fe(III)tetrakis-2-(*N*-triethylene glycol monomethyl ether)-pyridyl porphyrin (FP15) and Fe(III) tetra-mesitylporphyrin octasulphonate (FeTMPS) improve nerve function in streptozotocin-diabetic rats and streptozotocin-diabetic and *ob/ob* mice. The beneficial effects of the protein nitration inhibitor epicatechin have also been reported.

Multiple risk factor reduction has been shown to lower the hazard ratio for autonomic neuropathy by 63% [14]. What the important elements were in the Steno Type 2 study have yet to be determined. Even the maldistribution of cardiac sympathetic innervation can be restored with excellent diabetes control [1] [79].

An increase in heart rate variability has also been described with the use of aldose reductase inhibitors, C-peptide, ACE inhibitors (quinapril, ramipril, perindopril), angiotensin receptor blockers (losartan, telmisartan), cardioselective β-blockers without intrinsic sympathomimetic activity (e.g. metoprolol), digoxin and verapamil [1]. Autonomic imbalance and high post-infarction morbidity and mortality are frequently observed in patients with diabetes. Because the reduction in both recurrent myocardial infarction and mortality in post-infarction patients treated with β-blockers without intrinsic sympathomimetic activity was higher in subjects with diabetes than subjects without diabetes, it has been suggested that this high-risk group could particularly benefit from these agents.

To date, no results are available for advanced glycation end-product inhibitors (AGEIs), statins, carnitine, peroxisome proliferator activated receptors (PPARs), protein kinase C-β inhibitors and anti-inflammatory agents and the effect on heart rate variability. These agents are therefore some of the suggested targets for future attention.

**FIGURE 3:**
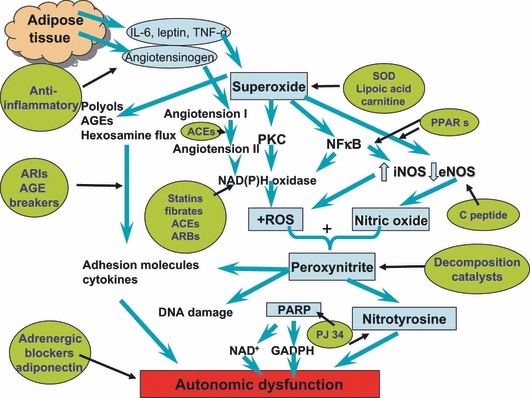
Suggestions of pharmaceutical approaches to the treatment or prevention of autonomic dysfunction in diabetes. Central to this evolving concept is the role of adipocytokines and inflammation. AGE, advanced glycation end product; ARB, angiotensin receptor blocker; ARI, aldose reductase inhibitor; e-NOS, endothelial nitric oxide synthase; IL-6 interleukin 6; i-NOS, inducible nitric oxide synthase; NFkB, nuclear factor-kappa B; PKC, protein kinase C; PPAR, peroxisome proliferator activated receptor; ROS, reactive oxygen species; SOD, superoxide dimutase; TNF-α, tumour necrosis factor alpha; Adapted from Vinik *et al*. (2006) with permission from the authors and the publishers of *Vascular Pharmacology* [46].

A paradigm of pharmaceutical approaches to reducing inflammation and oxidative/nitrosative stress is illustrated in Fig. 3 modified from that previously published [46].

## Summary

Autonomic imbalance between the sympathetic and parasympathetic nervous system's regulation of cardiovascular function contributes to significant morbidity and mortality for individuals with diabetes. Obesity and the overproduction of inflammatory cytokines have been implicated in the induction of numerous pathogenic mechanisms that can be responsible for autonomic imbalance impacting deleterious processes that increase and perpetuate cardiovascular risk. In addition, hyperleptinaemia and a deficiency of adiponectin favour sympathetic overactivity and the inflammatory cascade is enhanced in the presence of sleep apnoea, a frequent partner in diabetes and the metabolic syndrome. Restoration of autonomic balance is possible and has been shown with therapeutic lifestyle changes, increased physical activity, β-adrenergic blockers, aldose reductase inhibitors, ACE inhibitors, angiotensin receptor blockers, potent antioxidants such as α-lipoic acid, and in animal models using inhibitors of peroxynitrite formation and its decomposition. There are exciting new prospects for pathogenesis-oriented intervention.
